# The prosody of cheering in sports events: the case of long-distance running

**DOI:** 10.1515/phon-2024-0034

**Published:** 2025-12-19

**Authors:** Marzena Żygis, Sarah Wesolek, Nina Hosseini-Kivanani, Manfred Krifka

**Affiliations:** Leibniz-Centre General Linguistics, Berlin, Germany; Humboldt University, Berlin, Germany; University of Luxembourg, Belval, Luxembourg

**Keywords:** cheering contours, acoustics, speech acts, motivational speech

## Abstract

Since cheering has not yet been systematically investigated from a phonetic perspective, this study explores its acoustic characteristics by focusing on a specific type of cheering: inciting calls directed at individual runners during long-distance races, using their names. We investigate its prosodic realization through an experimental approach. We present findings from a production study with recordings in the lab comparing cheering utterances to neutral speech. 30 native speakers of German were asked to cheer on an individual marathon runner in a sporting event shown in a video by calling out his or her name (1–5 syllables). For reasons of comparison, the participants also produced the same names in isolation and carrier sentences. Our results reveal four different cheering patterns: (i) separately produced items of similar duration, (ii) division of items into syllables, (iii) mixed pattern of (i) and (ii), and finally (iv) a singing pattern, again mixed with (i) and (ii). When cheering for the marathon runners, participants used a higher fundamental frequency, a wider F0 range, longer item duration, slower speech rates, and increased intensity.

## 1 Introduction

In sporting events, various forms of cheering are frequently employed to motivate participants and enhance their performance. For instance, during marathons, spectators may chant phrases such as “Go, go!” “Faster, faster!” or specific names like “Alice, Alice, Alice.” Cheering is an activity of spectators and can be addressed to individuals or teams, depending on the sports event. It can be produced by individual cheerers, as is often the case in marathons, or by groups of fans, as in team sports like football. The focus of the present investigation is specifically on the former case: individual cheerers cheering for individual marathon runners.

What is cheering? To define cheering from a pragmatic point of view, one of the earliest mentions of cheering is found in Austin’s (1962) classification of speech acts based on performative verbs. He discussed only one cheering term, that is, “to toast”, which expresses a wish, which Austin classifies as a “behabitative” – in particular, an expression “of attitudes to someone else’s past conduct or imminent conduct.” In Searle’s (1969) classification of speech acts, cheers fit the class of expressives that communicate a psychological state. Searle’s closest example to cheering is “to congratulate”, exemplified by “I congratulate you on winning the race”. However, cheering is often directed at individuals involved in events that are still unfolding, with the purpose of encouraging them to achieve their goals in the first place. Also, cheering is not just an expression of the speaker’s psychological state but has the goal of supporting and encouraging the addressee (a dimension that is neglected in Searle’s class of expressives in general). Searle also mentions, in a footnote, the example “Hurrah for Manchester United!”, but only to illustrate that speech acts do not have to be clausal but can be based on a referring expression. Later classifications of speech acts were sometimes more specific about speech acts like expressives and desires but did not identify cheering as a separate act (cf. Kissine 2013 for an overview).

The main addressee of cheering in sports appears to be the athlete or team of athletes that the cheerers want to support in their ongoing efforts. As McCormick et al. (2015) state in a meta study, “verbal encouragement (…) can have a beneficial effect”. This point is illustrated in a complaint from a 1948 sports newspaper (as pointed out by a reviewer): “Due to the fact that cheering is an all-important function of a football game, we hope that in the future the cheerleaders will select the right cheers at the correct psychological moment” (*The Bates Student*). But cheering also creates a bond between the cheerers. Ludwig (2020) mentions cheers as one of the types of speech acts in which individual acts can be collectivized. Cheering is also one of the few speech acts that can be produced simultaneously by several people, not serially (Kerrison 2018; Lerner 2002). It can be classified, similar to booing, moaning, and laughing, as a choral phenomenon, often used for evaluation or response (see e.g. Tekin 2023).

There are a few observations on the function of cheering in sports that also address the effect on the cheerers themselves. For example, Bicknell (2011) looks at the “embodied relationship between athletes and cheering”, stressing the fact that cheering helps the spectators to join in or participate with the athletes, thus experiencing the same emotional struggle as the other spectators and the athlete. Without this, it would make no social sense to cheer when watching sports broadcasts, as the addressees cannot perceive the cheers. Hence, cheering has an expressive component, similar to exclamatives.

Cheers can be expressed in a multitude of ways. For instance, assertions like *You will make it!*, imperatives like *Go on!*, prohibitives like “*Don’t give up!*”, adhortatives like “*Let’s go!*” and non-finite forms like “*Faster, faster!*” can be used in English. There are dedicated cheering interjections like “*hurrah*” or earlier “*huzza*”, which can be traced back to the 16th century and which the Oxford English Dictionary (OED) describes as “a shout expressive of exultation, encouragement, or applause; used esp. as a ‘cheer’ in public assemblies of the like”. There are idiomatic cheers like the established chants of fan clubs, and non-verbal cheers like rhythmical applause, hands clapping, thumbs-up gestures, collective performance of waves, and showing support by dress or mascots.

Cheers that use names are different from calls (summons), as they are not just identifying the referent as addressee, see e.g. Pierrehumbert (1980), Ladd (2008), and Arvaniti et al. (2016) for an overview. In fact, it would be counter-productive in a race to call out for a runner, as this would probably distract them. Cheers are furthermore different from addresses (cf. Zwicky 1974), which identify the social and emotional relationship between the speaker and addressee. Addresses like “my dear”, “honey” or “Professor Smith” that express such relationships cannot easily be used for cheers. The term *cheering* in its natural use is rather unspecific; the OED lists 16 senses for the verb “to cheer”. In particular, it can be used for the support of ongoing events (“cheer someone on”), or for the expression of joy about recent achievements. It can also be used to express consolation about past failures (“cheer someone up”). German makes a rather consistent distinction here with the lexical items *anfeuern* (lit. “fire up”), *jubeln* (“rejoice”) and *aufmuntern* (roughly, “inciting”, “rejoicing” and “cheering up”).

It is the first meaning of cheering, also understood as the communicative support of ongoing events, that is a subject of investigation of the present paper. We aim to scrutinize how German speakers motivate marathon runners to win the competition. More specifically, we are interested in the prosodic patterns that are used by speakers to motivate the runners. As will be discussed below in Section 2 there are no studies that investigate this type of speech act from an (experimental) phonetic point of view. The most related studies seem to be Tekin (2023) and Kerrison (2018) who discuss cheering phenomena in the framework of conversation analysis, see Section 2 for details. Therefore, this investigation aims to fill in this gap and provide the first investigation of this topic.

The paper is organized as follows. In Section 2, we present an overview of previous phonetic studies related to the topic under investigation, focusing on motivational, charismatic, and emotional speech, all of which share some characteristics with cheering. In Section 3 our experiment including experimental design, measurements, statistical modelling and results is presented. Section 4 is devoted to the discussion of the results. Section 5 concludes.

## 2 Previous studies related to cheering

Since cheering has not yet been systematically investigated from a phonetic perspective, this section provides an overview of related speech types − such as motivational, charismatic, emotional, and Lombard speech − that may share acoustic features with cheering. At the end of the section, we also discuss two studies that address collective cheering from a descriptive standpoint (Kerrison 2018; Tekin 2023).

Motivational speech describes an act in which an individual motivates the interlocutor toward a specific action (Niebuhr et al. 2020; Skutella et al. 2014; Voße et al. 2022). The (acoustic) patterns that make speech motivationally successful have already been studied in a few investigations. For instance, Voße and Wagner (2018) inspected acoustic parameters of motivational speech, such as the speaking rate (syllables/second), F0 (high median, range, and variation coefficient) and intensity (high median, range, and variation coefficient), for each Interpausal Unit (IPU) that was perceptually stated in 6 Youtube videos. A categorization of less and more successful motivation was performed based on the videos’ online ratings by viewers. The acoustic investigation revealed that a more motivational speech is produced with a higher but less variant speaking rate, higher F0 and more variant F0 patterns, and higher intensity. At the same time, inconsistent results for the variation of intensity were observed, suggesting a more fine-grained analysis of this parameter.

Motivational speech was also a research subject in a study by Skutella et al. (2014), in which the prosody of interactions between an instructor and a trainee was studied during a session of an indoor cycling class. The study revealed that the instructors’ prosody contained a high number of prominent accents that were executed employing a strong lengthening and locally raised intensity. Additionally, the phenomenon known as stress clash, where two stressed syllables occur consecutively without an intervening unstressed syllable, a pattern typically avoided in speech, was frequently observed. Based on visual inspections of the data, the authors also point to prosodic iconicity, i.e. locally raised intonation, leading to high tones, mostly accompanied by upward movements, whereas locally falling intonation leading to low tones was produced in co-occurrence with downward movements. The results also show rhythmically structured pauses that were assumed to be used to synchronize verbal expressions with the individual steps of the cycling movements (note that no measurements linking verbal expressions and body movements were undertaken).

In another study, Paulmann et al. (2018) investigated how acoustic features (intensity mean and range; pitch mean and range; and speech rate) define different types of motivational speech. The materials consisting of 104 video clips with parent-child interactions in Dutch were perceptually judged for their motivational quality. In line with the Self-Determination Theory (Ryan and Deci 2017), controlling (“you must do this”) versus autonomy-supportive (“you may do this if you choose”) voices were selected for the acoustic analysis. Results revealed that intensity and speech rate significantly differed for both types of motivational speech. Controlling messages were produced with a louder voice and higher speech rate in comparison to the autonomy-supportive voice. Controlling prosody was also expressed with a smaller pitch range. Finally, no significant difference in mean pitch was found between both prosody types which let the authors conclude that pitch is not the driving force to convey different motivation types.

Furthermore, in a more recent paper by Voße et al. (2022), voice-quality features (and pragmatic aspects) of German motivational speech were investigated, again based on YouTube videos. They were classified into more versus less motivational speech (based on the number of views on the YouTube platform): three videos (35 min in total) represented a higher level of motivation and three videos (18 min in total) represented a lower level. In total, about 53 min of speech material were analyzed. The results point towards motivational speech being characterized by a more periodic signal indicating a fuller voice. In addition, the results regarding H1-H2 indicate reduced and less variable breathiness in motivational speech.

Motivational speech is closely linked to charismatic speech. Voße and Wagner (2018) understand charisma and motivation as intertwined concepts that share phonetic features. Concerning charismatic speech, the study conducted by Niebuhr et al. (2020) which examined speeches by Steve Jobs and Mark Zuckerberg concluded that the same tone-of-voice settings that make religious or political leaders sound more charismatic also work for business speakers. Classified as more charismatic, speeches delivered by Steve Jobs were characterized by a higher F0 level, a larger F0 range, a higher intensity level and fewer disfluencies. Also, prosodic phrases and intended silent pauses were shorter. It was also found, contrary to expectations, that Steve Jobs spoke significantly slower than Mark Zuckerberg, however, still faster than an average American speaker (Niebuhr et al. 2020: 26). Therefore, the comparison was performed in fact between a fast (Steve Jobs) and a very fast speaker (Marc Zuckerberg). Finally, also contrary to expectations, no intensity variability difference was found between the two speakers.

The findings by Niebuhr et al. (2020) are in line with previous research on charismatic speech that was characterised by higher F0, larger F0 range, higher intensity, higher speech rate, fewer disfluencies, shorter (silent) pauses and shorter prosodic phrases, see Rosenberg and Hirschberg (2009), D’Errico et al. (2013), Niebuhr et al. (2016), Berger et al. (2017), Bosker (2021), Mixdorff et al. (2018), Niebuhr and Ochoa (2019), Niebuhr and Firscher (2019), Niebuhr and Skarnitzl (2019).

Another type of speech that is also related to cheering is emotional speech which shares its features with motivational and charismatic speech types such as higher F0, a wider F0 range, a higher intensity, and a faster speech rate, see e.g. Banse and Scheerer (1996). Kamiloğlu et al. (2020) conducted a review of 108 studies investigating acoustic features associated with positive emotions. Their analysis revealed that vocal expressions of happiness are generally characterized by increased loudness with substantial variability, higher and more variable F0, and elevated frequencies in the first two formants. By contrast, negative emotions such as sadness are usually characterized by a low F0, lower amplitude, and a slow speech rate. Despair is marked by an even slower speech rate than sadness, with greater F0 variability. Panic fear, on the other hand, is produced with a very high F0, high energy, and a fast speech rate, reflecting heightened arousal (Banse and Scheerer 1996; Scherer 2003).

The study by Trouvain and Barry (2000), which examined horse race commentaries, is linked to cheering by the amount of emotion expressed in connection with a competition. The authors analyzed three commentaries lasting from 79 s (race 1) to 145 s (race 3), produced by speakers of Standard Southern English, New Zealand English, and Australian English. They found that commentators exhibited a higher average F0, a wider F0 range, and greater intensity, but a varying speech rate when they appeared to be more emotional and motivational (typically towards the end of the race).

Apart from motivational, charismatic and emotional speech, it seems that due to the noisy context of sports events, the prosodic features of cheering might be related to that of Lombard speech (Lombard 1911). This is a phenomenon where individuals unconsciously raise their voices in response to elevated ambient noise levels. This adjustment helps speakers maintain effective communication in noisy environments (Applebaum and Hanson 1990, Kobashikawa et al. 2019). As has been widely shown, the Lombard effect is not only characterised by increased amplitude but also by increased fundamental frequency, a longer duration (of vowels), reduced speaking rate as well as a shift in formant frequencies for F1 and F2 (Anglade and Junqua 1990; Applebaum et al. 1996; Hanley and Steer 1949; Hansen 1988).

There are two studies that we are aware of that investigated collective cheering. The first is a paper written in the ethnographic fieldwork of Tekin (2023). The author observed and videotaped people playing a video game, specifically a bowling game on an Xbox console equipped with Kinect, a motion-sensing device. The study includes 20 h of video data. Some descriptive observations of choral vocalizations listed by the author are the elongation of vocalizations as an invitation for other people to join the cheering, the theatrical way performed in which players encourage choral vocalizations and the modification of the vocalizations in relation to an event occurring in the game (e.g. change of vocalization from [oː] to [aː] marking a recognition of a changing event).

Kerrison (2018) investigated sports fan cheering as a collaborative undertaking. He examined video data of informal cheering groups at ice hockey contests by using conversation analysis. The goal of the analysis was to see how the turns of the cheering event orient to the turns of play. As Kerrison (2018: 282) stresses, the study provides a language and structure to begin discussing cheering as a type of talk-in-interaction, with its constraints of turn-construction compared to conversation. He also identified and discussed cheering practices that he categorized into four general structural forms, i.e., *response cries* (yells, cries, groans, growls and yelps directed at one’s own state, e.g. “oops”, Kerrison 2018: 222), *response clusters* (the iconic crowd responses, the roars of the crowd, applause and booing including a long obstructive “oh” -ing), and *chanting* (including a chanting cadence where both syllables and pauses are progressed at the same rate, Kerrison 2018: 240), and *routines* that are most complex (individually learned forms that considerably vary, e.g. rhetorical questions).

Finally, it is also important to differentiate between general cheering as described above, i.e., the verbal encouragement offered by spectators, and the structured activity of cheerleading. While cheering is a spontaneous expression of support, cheerleading has evolved into a formalized practice that combines dance, acrobatics, and chants. This transformation reflects broader cultural shifts, particularly in the United States, where cheerleading has become both a symbol of school spirit and a competitive sport in its own right. Interestingly, what is now widely perceived as a predominantly female activity, actually originated as a male-dominated practice in the early 20th century. For example, Franklin D. Roosevelt, who would later become President of the United States, participated in cheerleading during his youth; see also Grindstaff and West (2006).

To sum up, despite numerous studies on motivational, charismatic, emotional, and Lombard speech, there are no acoustic investigations of cheering produced by individuals. The findings from these areas, however, suggest that cheering might share key prosodic characteristics with other expressive speech types, such as, increased F0, wider F0 range, greater intensity, and slower or rhythmically structured timing. These insights from the existing literature form the basis for our hypotheses presented in the next section, where we test whether cheering indeed exhibits these prosodic features and how they are realized in individual cheering calls.

## 3 Experimental evidence

Following our research goal, we investigate the prosodic patterns of inciting cheers in a scenario that can be found in a marathon event when individuals are cheering on single runners. We analyse how the cheerers employ different acoustic parameters including duration, amplitude, F0, F0 range and speech rate to achieve their goals. We compare the cheering items with corresponding items produced in a neutral context, i.e. appearing in isolation and sentences. Such a design will make a comparison with items uttered in other contexts more straightforward.

Based on Voße & Wagner’s (2018) study on charismatic speech, we hypothesized names in the cheering context would be produced with a higher pitch than names pronounced in isolation and frame sentences. A higher pitch might also evolve as an expression of high arousal emotions, see Bänziger and Scherer (2005). Similarly, we expected the F0 range to be larger in the cheering context than in other contexts. Cheering items will be, similar to charismatic speech (Voße and Wagner 2018), louder than names produced in the neutral context. Additionally, studies have shown emotions to be expressed and detected, besides others, by the psychoacoustic feature of higher loudness (see, e.g. Coutinho and Dibben 2013). We also hypothesized cheered utterances to be shorter as they may align more with the high speed of the runners during the marathon. For the same reason, the cheering speech rate was expected to be faster.

In summary, our research hypothesis is as follows:

**Names produced in a cheering context are shorter, louder, have a higher F0 and a wider F0 range, and are spoken at a faster (within-a-phrase) speech rate than names produced in a neutral context − both in isolation and in sentences**.

### 3.1 Experimental design

To test our research hypothesis, we conducted an acoustic experiment divided into three blocks. In the first block, German-speaking participants were asked to produce male and female names in isolation three times in a randomized order. In the second block, they were instructed to pronounce the same names embedded in a frame sentence (*Ich habe X gesagt*, “I said X”), repeating them again three times in a randomized order. We used these two blocks as we were expecting differences, especially in F0 which should be flatter for names embedded in sentences and rising for names produced in lists. We also aimed to see whether the cheering items would be produced similarly to embedded or isolated names. In the third block, participants were asked to produce the same names in a cheering mode, while watching a typical marathon video. They were instructed to imagine that one of their friends was running a marathon and that they should cheer them on by using their name. There were two types of videos: one featuring male runners (with male names) and another featuring female runners (with female names). Using two recordings was unavoidable because the videos were authentic recordings of marathons/running competitions, in which participants are typically either all male or all female (see Section 4 for minor differences in background noise between the videos). In each video, the highlighted runner was marked with a moving light to facilitate tracking. In the left bottom corner of the video, the name of the runner was displayed, see Figure 1. During the cheering task, participants wore over-ear headphones and listened to noise and expressions (e.g. *go, go, go*) and clapping typical for the situation. The background noise was not recorded. Importantly, in this block, there were three video series, each containing 10 short videos presented again in a randomized order. The duration of one short video corresponding to one male or female runner varied from 28 to 37 s. While in the first and second block, 60 names were produced (30 in each block), the number of the items in the third block differed, as participants were free to produce the items as many times as they wished. The number of produced name tokens varied from 138 to 680 per speaker in all three series pointing to individual differences. We ended up with the recordings of 12,900 tokens that were submitted for further analysis.

**Figure 1:**
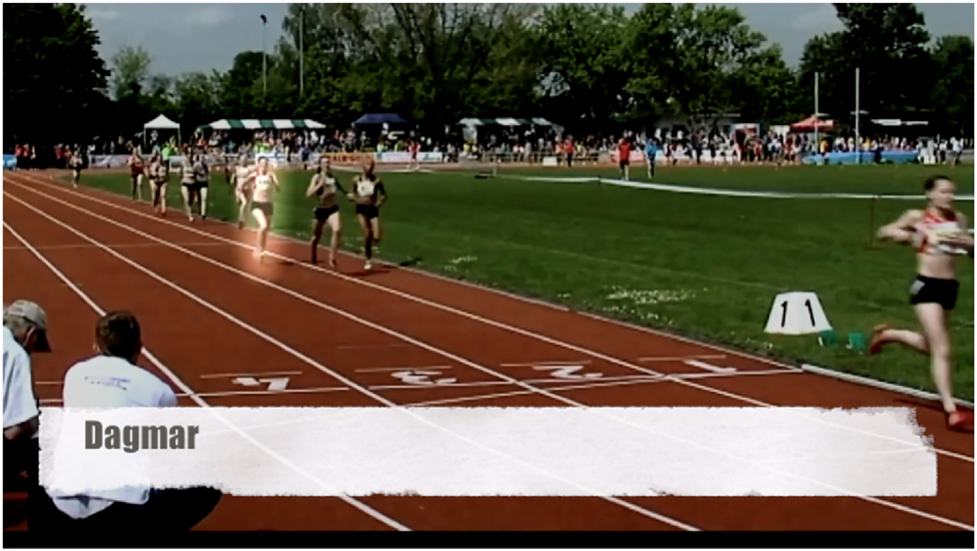
Screenshot of a video used in the experiment for the stimulus “Dagmar”.

The experiment took place in a sound-proof lab at the Leibniz-Centre General Linguistics (ZAS) in Berlin. Before starting the experiment, participants were informed about the order of presentation of the three blocks. Block 1 (items in isolation) and block 2 (items embedded in carrier sentences) were presented using PowerPoint and block 3 (videos with names) was shown with the VCL media player. The participants were recorded with a Sennheiser MKH 20 P48 microphone positioned ca. 20 cm from their mouths. The recordings were made with a sampling rate of 48 kHz and 16 bit. The whole experiment took 25 min on average.

The cheerers were assured that they would not be heard by anyone during the experiment enabling them to vocalize and emulate the behaviour of genuine sports enthusiasts. Post-experiment oral feedback revealed that several participants enjoyed their participation as it gave them an opportunity for uninhibited expression. Additionally, some participants noted that the experiment felt therapeutic for them. This provides indirect evidence that the participants were motivated and engaged themselves in the task.

### 3.2 Informants

31 native speakers of German took part in the experiment. Due to a damaged sound file from one participant, data from 30 informants underwent further analysis. There were 20 female and 10 male speakers aged 19–50 (mean: 30.51, sd: 10.27). The participants were recruited through LingEx, a registration site for linguistic experiments of the Leibniz-Centre General Linguistics (ZAS) and Humboldt-Universität zu Berlin. Consent forms and questionnaires about metadata were filled in by participants before the experiment, following the ethics approval for the ERC SPAGAD project (787929). One participant did not fill in the form about the metadata, so his/her background data are missing.

### 3.3 Materials

Our material consisted of ten words: five female and five male names, predominantly composed of sonorants. The number of syllables varied from one to five and the lexical stress was always placed on the penultimate syllables (apart from one-syllabic words). A prevailing number of syllables were open (CV). Table 1 presents the stimuli with their phonetic representation. Dots indicate syllable boundaries. Please note that not each item was phonetically realized by each speaker as shown in Table 1. For instance, *Emanuela* was sometimes pronounced with one or two glottal stops [ʔ] ([ʔe:.ma.nu:.ˈʔe:.la]), *Bartholomäus* with [e:] ([baɐ.to:.lo:ˈme:.ʊs]). Following a reviewer’s comment we also counted the number of Daniel and Daniela as three- and four-syllabic words respectively. It turned out that *Daniel* was pronounced 34 times (by three speakers in total) as a three-syllable word [ˈdaː.ni̯.ɛl]. This constituted 2.81 % of all *Daniel* realisations (1,206). *Daniela*, on the other hand, was produced 55 times as [daː.ˈni̯.eː.la] by six speakers, which constituted 5.41 % of all realisations of this word (1,016).

**Table 1:** Stimuli used in the experiment.

Female names		Male names		N. of syllables
Linn	[lın]	Jan	[jan]	1
**Dag**.mar	[ˈdaɡ̥.mɐ]	**Da.**niel	[ˈdaː.ni̯ɛl]	2
Da.**nie**.la	[daː.ˈni̯eː.la]	Jo.**han.**nes	[joː.ˈha.nǝs]	3
An.ge.**li.**na	[an.dʒɛ.ˈliː.na]	A.le.**xan**.der	[aː.lɛ.ˈksan.dɐ]	4
E.ma.nu.**e**.la	[eː.ma.nʊ.ˈeː.la]	Bar.tho.lo.**mä.**us	[baɐ.toː.loːˈmɛː.ʊs]	5

### 3.4 Measurements

The data were annotated and analysed in PRAAT (Boersma and Weenink 2019, version 6.1.08).

After a semi-automatic annotation, the segment boundaries in half of the recordings were manually checked by one annotator, and those in the other half by another annotator. Subsequently, the entire annotated dataset was double-checked by a phonetically trained annotator.

Employing scripts written for the purposes of this study, the following parameters have been measured:

– Durations of the words and all their syllables (in ms)
– Root Mean Square (RMS) amplitude of the words and all their syllables (in Pascal)
– F0 maximum and F0 mean of the words and all their syllables (in ERB)
– F0 range of words, i.e., a difference between F0 max and F0 min (in Hz)
– Speech rate: the number of syllables per second (syll/sec) in the inter-pausal unit (IPU)

While we expressed the F0 maximum and mean in ERB, we used Hz to measure the F0 range. This decision was motivated by the following considerations: Hz is a linear, physical scale that directly quantifies absolute frequency differences. When measuring the range of F0, our goal was to assess the extent of frequency variation, regardless of how these variations are perceived. Since the range is calculated as the difference between the maximum and minimum F0 values, it is practical to express it in Hz to represent the raw physical extent of variation. In contrast, ERB (Equivalent Rectangular Bandwidth) is a perceptual scale that better reflects how humans perceive pitch differences. Pitch perception is not linear in Hz; rather, it follows a logarithmic-like relationship. This means that the same frequency difference at higher Hz values is perceived as smaller compared to the same difference at lower Hz values. Using ERB for maximum and mean F0 measurements aligns these values with human perceptual sensitivity, offering more meaningful insight into how listeners experience pitch variations.

Before calculating the speech rate, we inspected the data and decided to identify the inter-pausal units (IPUs) that would serve as the domains for further calculations, such as speech rate. Since the cheering items were often produced as short series, our aim was to calculate the speech rate for each series, which often varied even within a single speaker. The IPU was defined by setting the pause duration to 2 s or more, based on our inspection of the data and measurements of pauses. Pauses of 2 s or longer ensured that items belonged to one larger prosodic unit. We then calculated the number of syllables per second within each IPU.

### 3.5 Statistics

The statistical analysis was conducted in R (R Core Team 2022) by using the packages *lme4* (Bates et al. 2018) and *emmeans* (Lenth 2019). We built separate linear mixed models with Duration, Amplitude, F0, F0 range and Speech rate as dependent variables, as described in Section 3.4. We tested how Context Type [items in isolation, items embedded in frames, cheering items], the Number of Syllables of names [1–5], participant’s Sex [male, female], grammatical Gender of Names [male, female], and participant’s Age influenced the dependent variables. We included the interaction of participant’s Sex and the grammatical Gender of names. We also added Item and Participant as random intercepts, as well as random by-Participant slopes for the Context Type, the Number of syllables, and a random by-Item slope for the Context Type. Due to convergence issues, some of the random slopes had to be removed. We started with full models, removed slopes that led to convergence issues and selected the final model. The models have also been checked with respect to (i) the normality of the residuals, (ii) their homoscedasticity and (iii) multicollinearity among the factors. To assess the significance of fixed effects in the final model, we conducted an Analysis of Variance (ANOVA) using Type III sum of squares. This approach tests each predictor’s unique contribution to the model while accounting for all other variables and their interactions. The Satterthwaite approximation was applied to estimate degrees of freedom, providing more accurate p-values in the presence of random effects. This method is particularly appropriate given the mixed-effects structure of our model and the unbalanced nature of the dataset.

Since the variables Context type and Number of syllables each had more than two levels, we conducted multiple pairwise comparisons between the levels. To control for the increased risk of Type I errors due to these multiple tests, we used a Tukey adjustment implemented through the emmeans() function.

For the analyses of duration and F0 range the data were log10-transformed because they were positively skewed. Nevertheless, we did not log-transform the data when visualising the profiles of individual token lengths (in the figures) to make a comparison for different contexts more transparent. The results we report are based on the measurements of 12,900 items. In three models where the dependent variable was log-transformed, extreme outliers were removed based on visual inspection (four 14 data points for duration and 13 for F0 range). We also tested removing outliers using a 3 standard deviation (3 SD) criterion, but in our view, this would have excluded too many data points. Additionally, some values were missing, mainly due to extraction errors. Overall, the following numbers of items were submitted to the final analysis: 12,876 items for *Duration*, 12,341 *Amplitude* , 12,900 for *F0,* 12,877 for *F0 Range* and 12,900 for *Speech Rate*.

Since we were also interested in which of the investigated variables (*Duration* , *Amplitude* , *F0* , *F0 range* and *Speech rate* ) is assigned a crucial importance for the selection of cheering contours we employed a random forest model designed to identify the most crucial variables, particularly in scenarios involving potentially collinear predictor variables (see Tagliamonte and Baayen 2012). The random forest comprised 2,000 individual trees and aimed to discern which parameters derived from the acoustic analysis (see Section 3.4.), contributed to the selection of cheering contours versus others (isolated, and embedded in sentences). For each tree, prediction accuracy was recorded based on the out-of-bag portion. This process was iterated after permuting each predictor variable. Ultimately, the difference between the accuracies was averaged over all trees and normalized by the standard error, following the methodology outlined by Kuhn (2019). To conduct this analysis, we utilized the “party” package (Strobl et al. 2008) and the “caret” package (Kuhn 2019). Scripts for statistical modelling, the database as well as codes for figures are available on an OSF account: https://osf.io/rkxdf/. The HTML document (https://osf.io/rd2hq) also includes tables with estimates, standard errors, degrees of freedom, t-values and p-values.

### 3.6 Results

Our results show that names produced to cheer on runners are produced as sequences of names differing in their arrangement. After careful listening to the data of all speakers, four main patterns were detected. Thus, speakers produced the names with:

(1) longer pauses between the items (the first pattern, see Figure 2)
(2) divided into syllables (the second pattern, see Figure 3)
(3) longer pauses intertwined with item sequences produced without pauses (the third pattern, see Figure 4). This pattern includes both previous patterns.
(4) with a melody, often prolonged syllables and several pitch peaks (the fourth pattern, see Figure 5)

**Figure 2:**
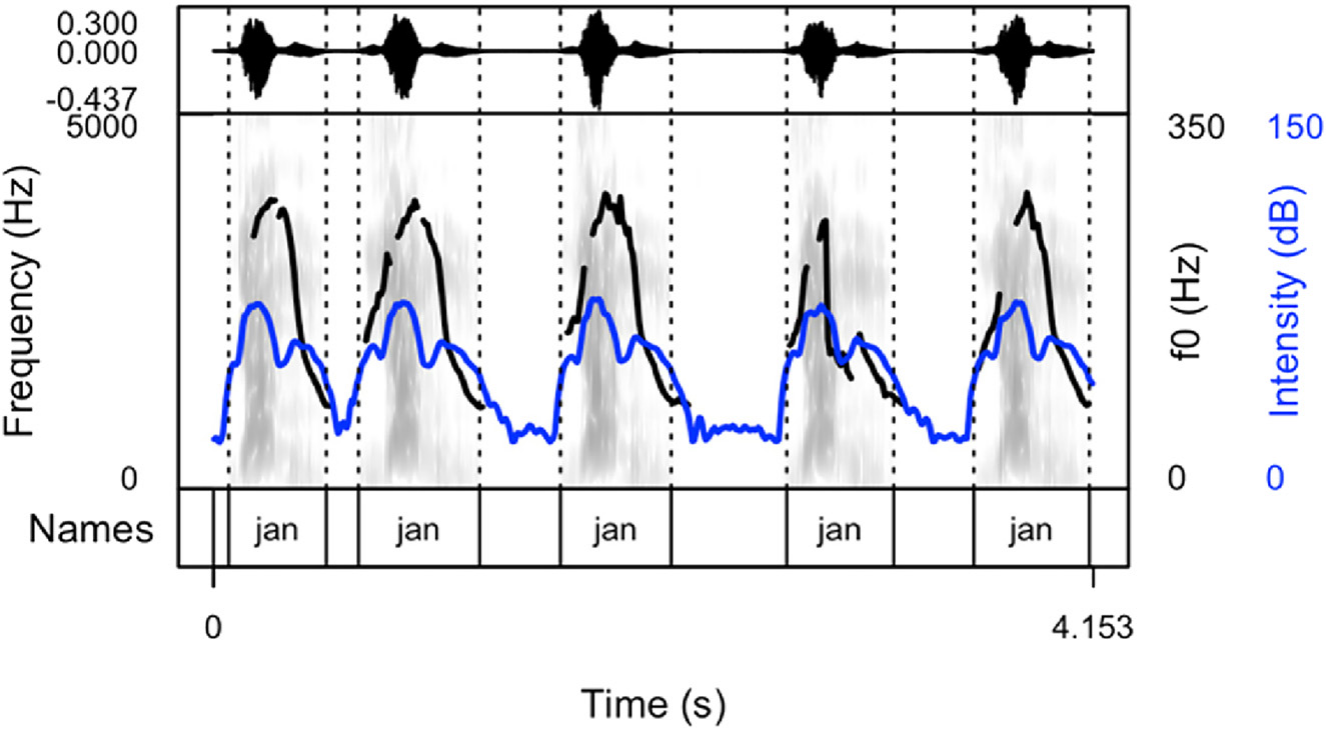
Spectrogram and oscillogram of a typical production of the name “Jan” in a cheering context (pattern 1).

**Figure 3:**
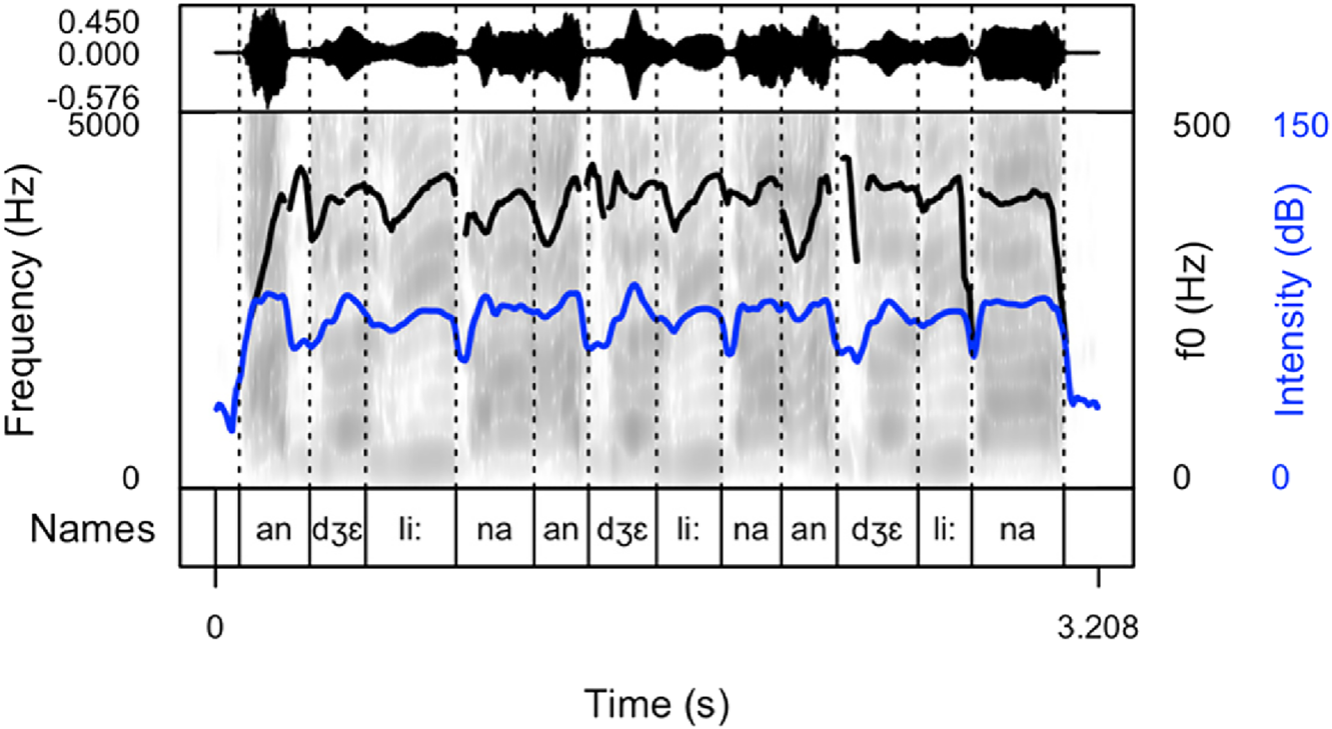
Spectrogram and oscillogram of a typical production of the name “Angelina” in a cheering context (pattern 2).

**Figure 4:**
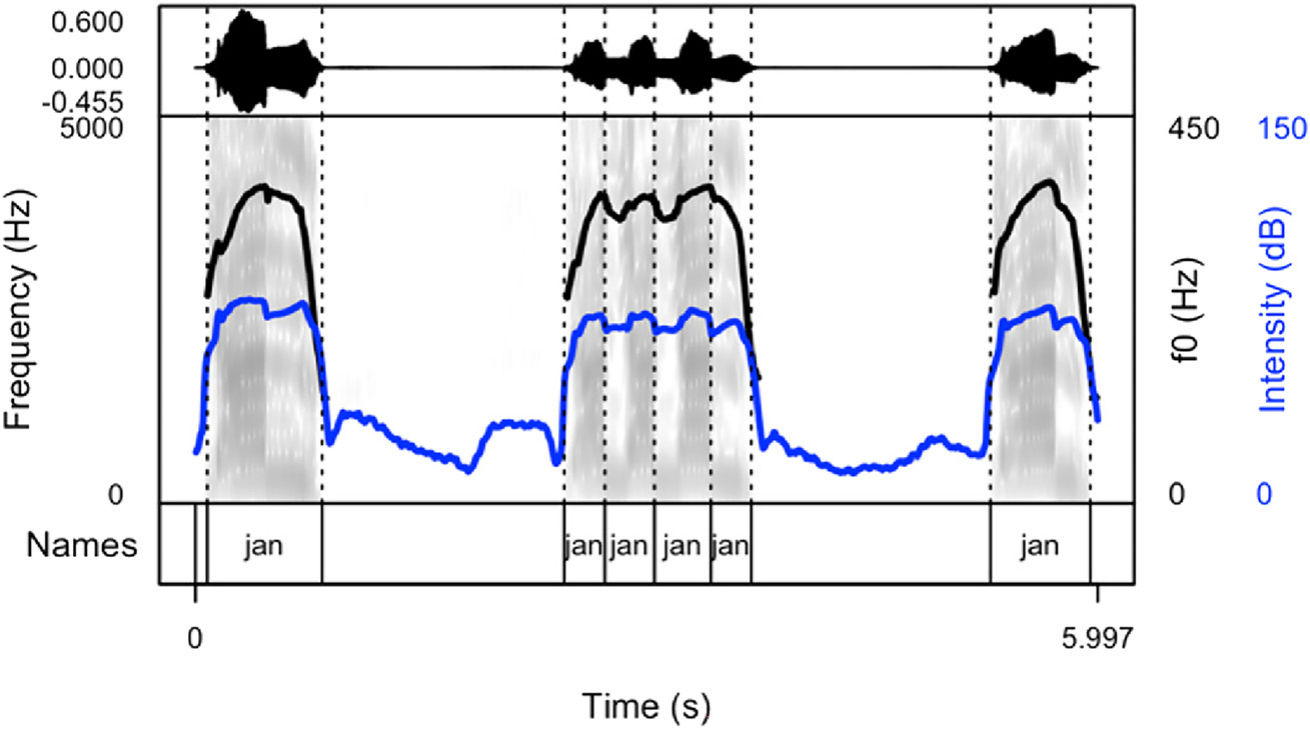
Spectrogram and oscillogram of a typical production of the name “Jan” in a cheering context (pattern 3).

**Figure 5:**
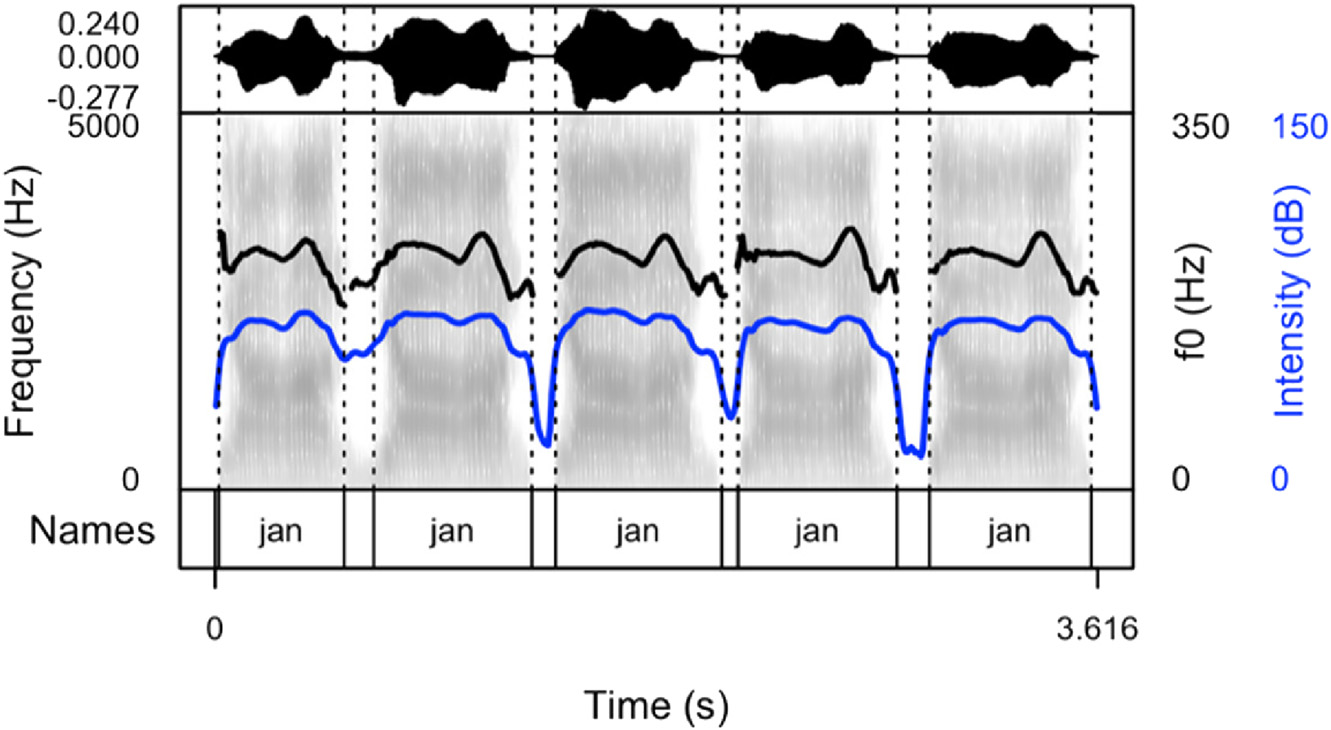
Spectrogram and oscillogram of a typical production of the name “Jan” in a cheering context (pattern 4).

The following figures provide examples of the cheering patterns. The examples of items produced in isolation and in sentence frames are presented in the Appendix (Figures 1A and 2A). Please note that all figures were prepared by means of the *praatpicture* library (Puggaard-Rode 2024). In the figures below (Figures 2–5), the black (upper) line indicates F0, and the blue (lower) line shows intensity.

The analysis of cheering contours indicates that 13 speakers consistently employed pattern 1, characterized by the generation of longer items accompanied by relatively extended pauses. Conversely, 2 speakers exclusively adopted pattern 2, resulting in the segmentation of multi-syllabic names into syllables of comparable duration. Notably, these speakers aimed for rapid production with perceptible divisions between syllables. In some instances where names comprised three or more syllables, a noticeable shift in lexical stress occurred, emphasizing the initial syllable (see Section 3.6.7). Moreover, 12 speakers exhibited a preference for a hybrid approach (pattern 3), illustrated in Figure 4. This group alternated between producing longer sequences of separate calls (pattern 1) and shorter, syllabified calls (pattern 2). Within this subset, 8 speakers favoured pattern 2 over pattern 1, while 4 speakers exhibited the opposite preference. Finally, in addition to employing the mixed form, speakers incorporated a melodic call, as depicted in Figure 5. In this pattern, even monosyllabic words were prolonged and featured two fundamental frequency (F0) peaks. This melodic pattern, in conjunction with previous patterns, was identified in the production of three speakers.

#### 3.6.1 Duration

**Figure 6:**
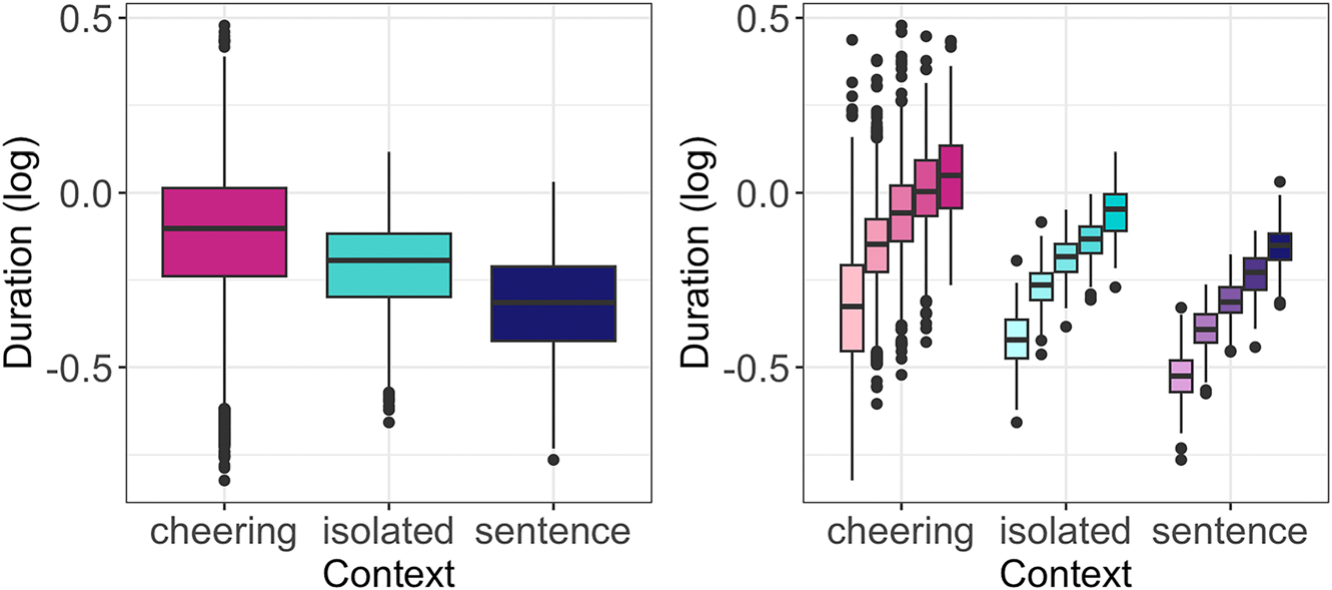
Duration of words in different contexts (left) split for 1- to 5-syllabic words (right).

The results reveal a significant effect of the context type on duration (F(2, 12,188) = 3,281, p < 0.001). In particular, cheering items were longer in comparison to items spoken in isolation (b = 0.127, t = 39.33, p < 0.001) and to items embedded in frame sentences (b = 0.239, t = 74.21, p < 0.001), see Figure 6 (left).1Please note that the order in which the results are presented (cheering, isolated, sentence) differs from the experimental order (isolated, sentence, cheering). This adjustment was made because the focus of the results’ presentation is on the cheering items, while the other items serve a comparative role. As expected, the duration of words was also dependent on the number of syllables they consisted of (F(4, 8.2) = 87.59, p < 001; p < 0.001 for almost all post-hoc comparisons), see Figure 6 (right) where the words were also additionally split for context. Female speakers produced female names shorter than male names, whereas male speakers did not make such a difference (interaction: F(1, 12,193) = 8.04, p < 0.05). The mean values were as follows: female speakers calling female names: 770 ms, female speakers calling male names: 790 ms, male speakers calling female names: 800 ms, and male speakers calling male names: 810 ms. The effect size was small (0.979 after conversion from log values), indicating that female speakers produced female names approximately 2.1 % shorter than male speakers producing male names. Age did not exert a significant difference in the duration of the names.

**Figure 7:**
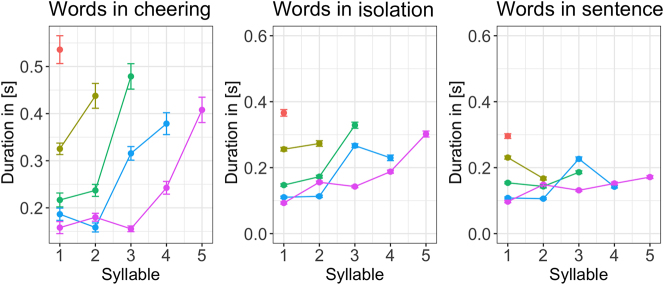
Duration of syllables for 1–5 syllabic words split for contexts.

Depending on the context, the individual syllables created profiles that differed in their duration. Figure 7 illustrates the duration of individual syllables in 1 to 5-syllabic words across different contexts. It is evident that longer words in cheering are characterized by increasing the duration of prefinal and final syllables. A similar conclusion applies to words produced in isolation, apart from a four-syllabic word where the final syllable is shorter than the prefinal one but still longer than the second one. A different pattern is found in words embedded in sentences where no such tendency is found. Please note that the figures are based on mean values and standard errors. For ease of comparison, they were not log-transformed. Also, no statistics were performed for these data. The line colours indicate a different word length.

**Figure 8:**
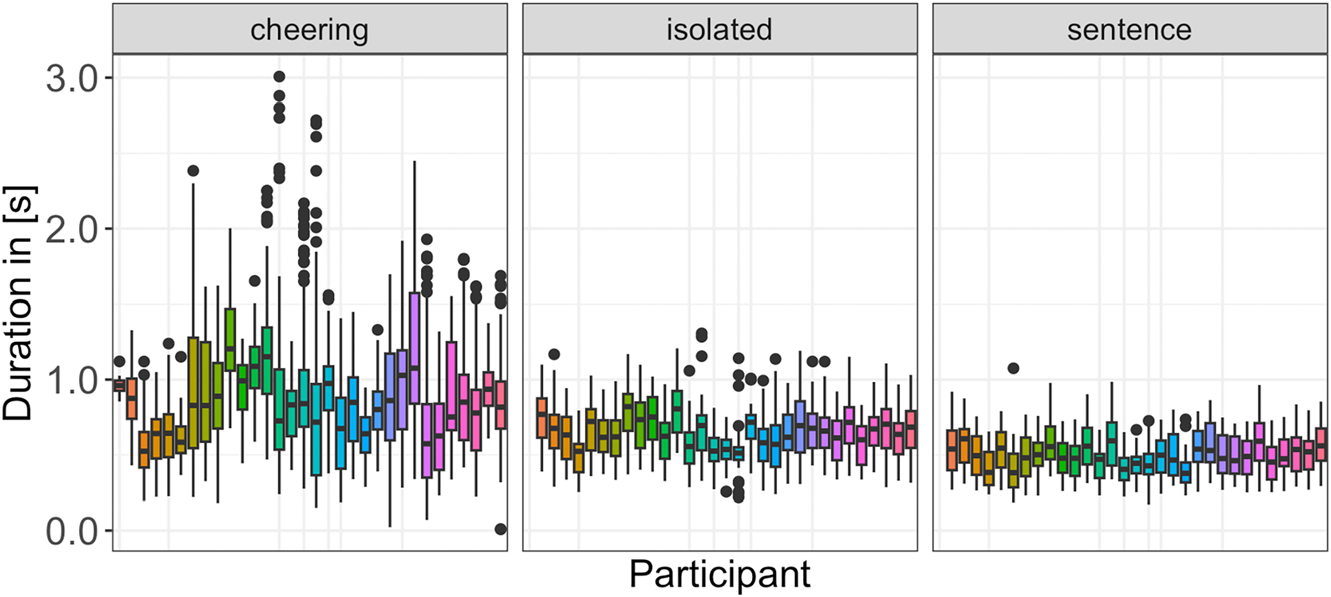
Duration of words in different contexts for individual participants.

Finally, we would like to point to an extensive interspeaker variation that characterizes items produced in the cheering context in comparison to other contexts. Figure 8 shows the results for each speaker individually across all three contexts. Here we also did not log transform the data and excluded 9 outliers.

#### 3.6.2 RMS amplitude

**Figure 9:**
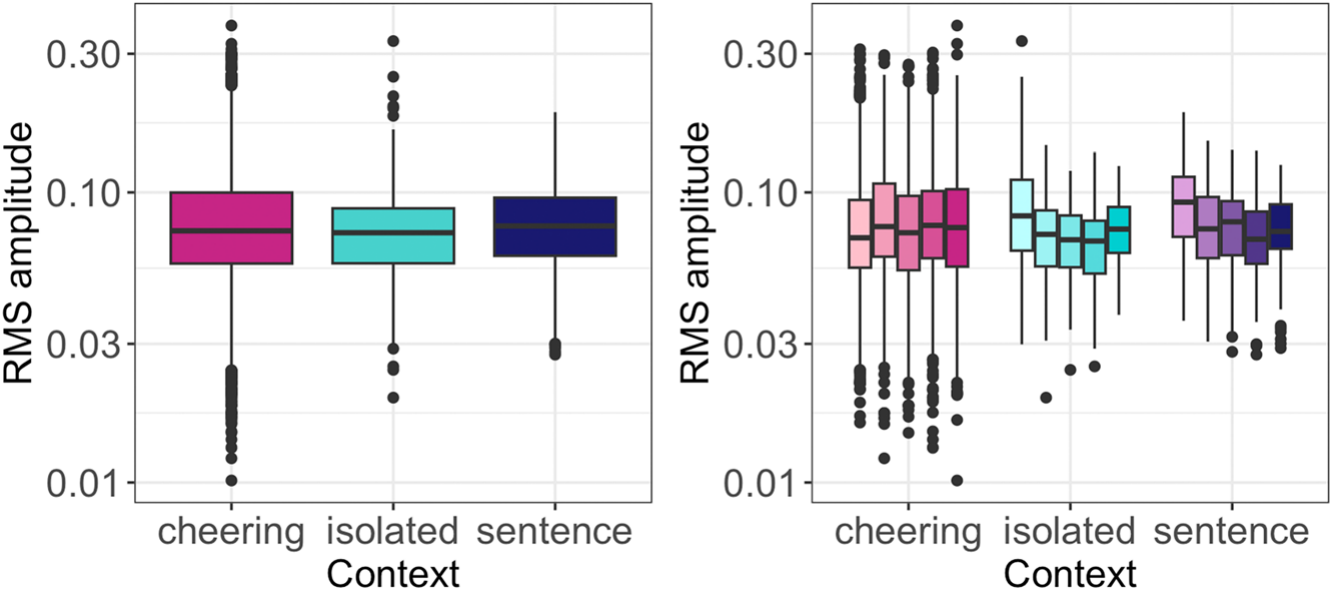
RMS amplitude of words in different contexts (left) split for 1- to 5-syllabic words (right).

Regarding the amplitude, the statistical modelling revealed a significant effect of the context type (F(2, 12,18) = 79.9, p < 0.001). Tukey-adjusted pairwise comparisons showed that items produced in the cheering context were louder than items produced in the isolated (*b* = 0.011, *t* = 11.01, *p* < 0.001) and sentence context (*b* = 0.007, *t* = 7.21, *p* < 0.001),2In Żygis et al. (2022), the observed differences were at the level of statistical tendency but did not reach significance. This discrepancy arises from our initial modeling, which employed amplitude measured in dB. In the present study, we transitioned to RMS amplitude, a more normalized measurement, to better capture and interpret the observed differences. see Figure 9 (left). Taken together, no significant differences were found depending on the word length. The one-syllabic words were not louder than two- and polysyllabic words, see Figure 9 (right) where the words are also split for context. Finally, male speakers were louder than female speakers (*F(1, 25.8)* = 5.60, *p* < 0.05). The influence of age was not significant.

**Figure 10:**
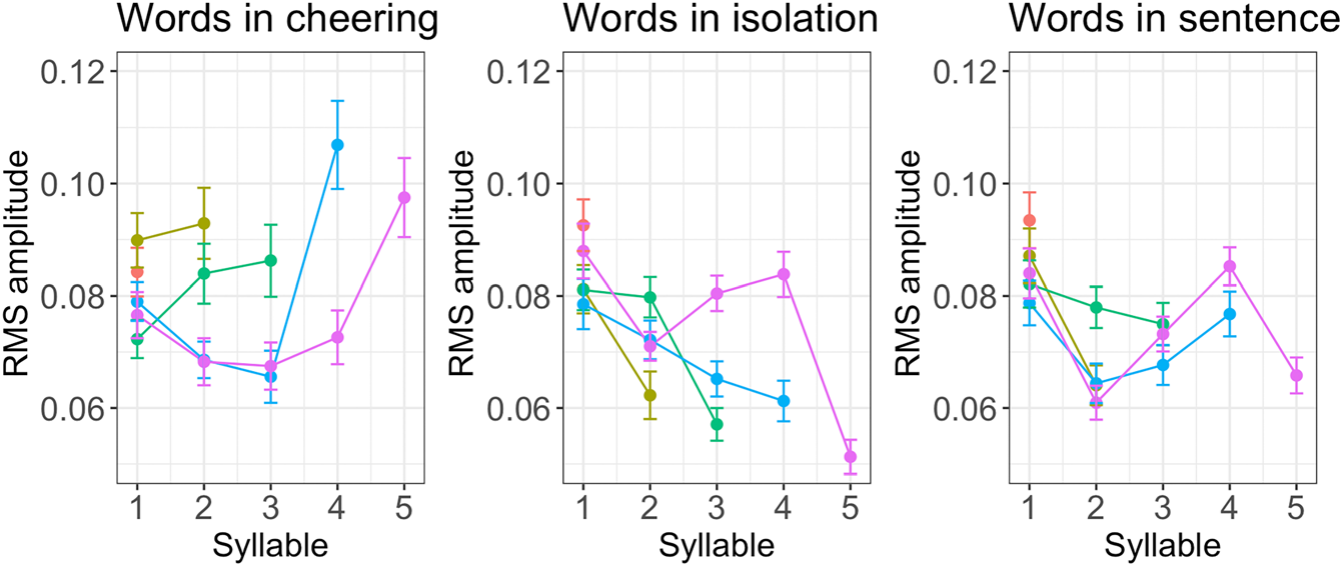
RMS amplitude of syllables for 1–5 syllabic words split for contexts.

Figure 10 illustrates the RMS amplitude of individual syllables in words of different lengths. It turns out that there is a considerable difference in RMS amplitude between the cheering context on the one hand and the isolation and sentence context on the other. What we observe is a steeper increase in the former, especially in the prefinal and final syllables, and a decrease in the latter contexts. The only exception to this pattern is four-syllabic words produced in sentences in which the amplitude in the final syllable was slightly increasing.

**Figure 11:**
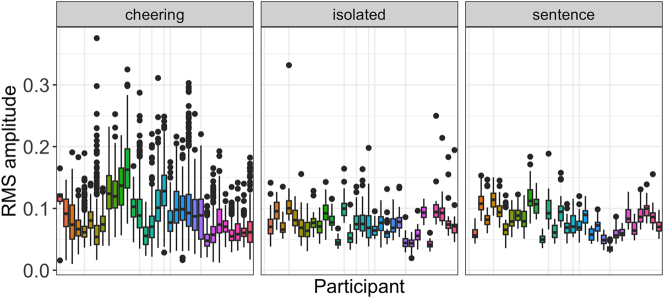
RMS amplitude in different contexts for individual participants.

What we can infer from the data is also its huge inter-speaker variation, especially in the cheering context, as illustrated in Figure 11. It is also evident that several speakers used a higher amplitude in the cheering context as compared to the isolation and sentence context.

#### 3.6.3 Fundamental frequency

**Figure 12:**
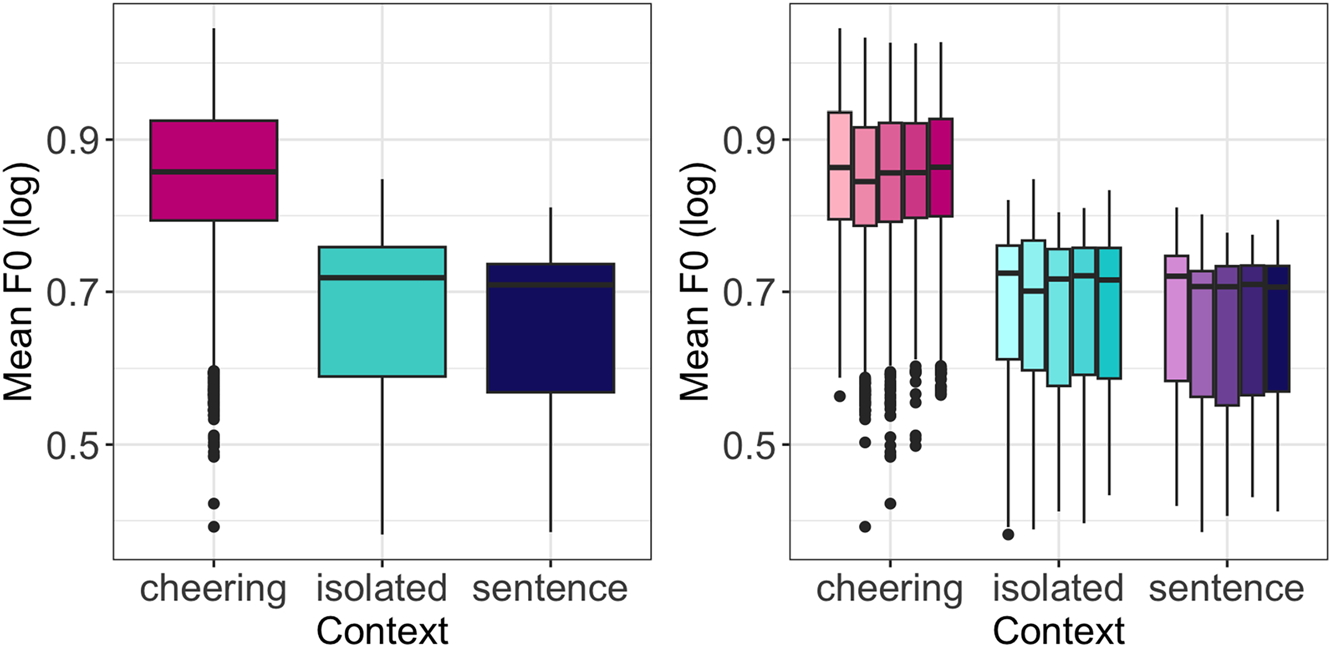
F0 of words in different contexts (left) split for 1- to 5-syllabic words (right).

A very clear picture emerges from F0 patterns. As in case of duration and amplitude, the influence of context type was significant (F(2, 12,271) = 13,512, p < 0.001). Speakers produced a significantly higher mean F0 when cheering as compared to their production of the same words in isolation (b = 0.18, *t* = 116.91, *p* < 0.001) and embedded in a sentence (*b* = 0.20, *t* = 125.97, *p* < 0.001), see Figure 12 (left). No significant differences were found between different word lengths. Figure 12 (right) shows the F0 split for word length and context. As expected, male speakers produced lower F0 than female speakers (*F*(1,28) = 20.96, *p* < 0.001). Age remained not significant.

**Figure 13:**
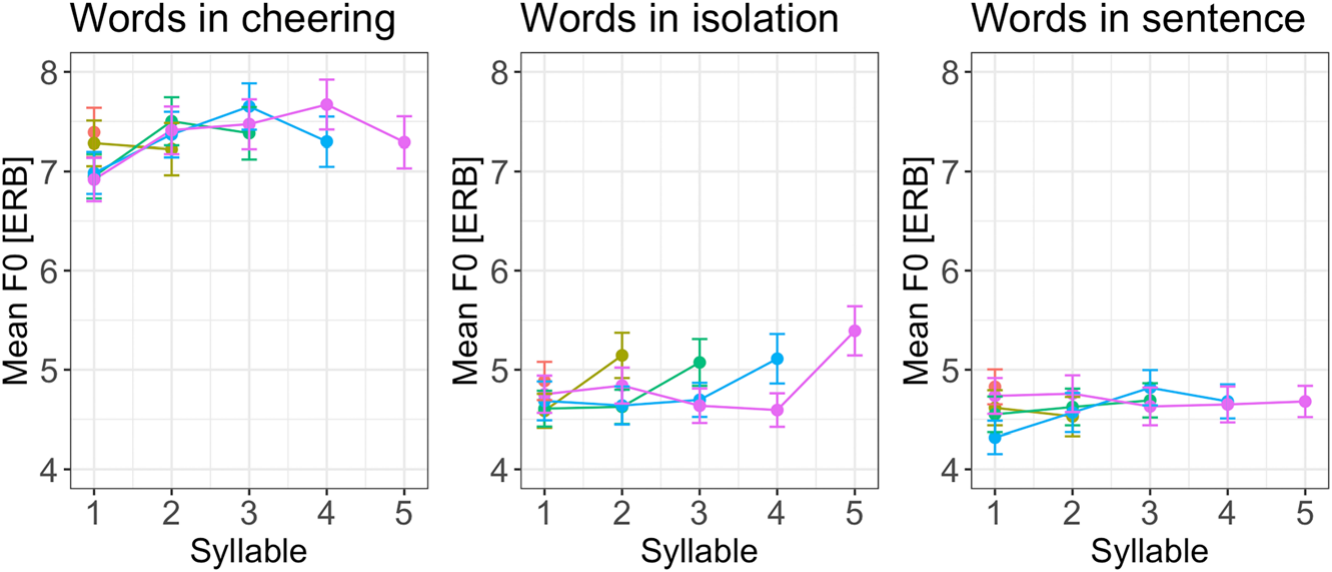
F0 of syllables for 1–5 syllabic words split for contexts.

If we consider F0 profiles for different word lengths in various contexts, it appears that from the penultimate to the ultimate syllable, F0 decreased in the cheering context, increased in isolation, and remained relatively flat in sentences; see Figure 13 provided here for illustrative purposes only (and not quantitatively analysed).

**Figure 14:**
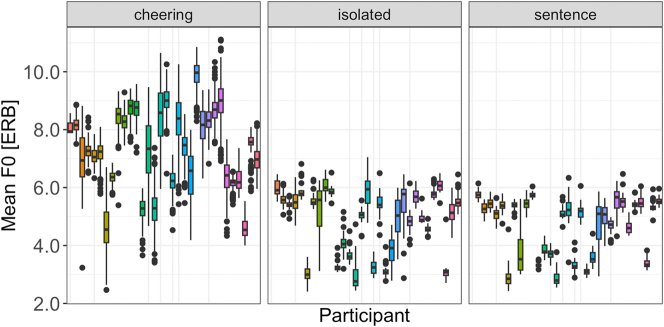
F0 in different contexts for individual participants.

As illustrated by Figure 14, the data show a considerable inter-speaker variation. However, it is found not only in cheering, as was the case concerning the duration and amplitude, but also in the isolated and sentence context.

#### 3.6.4 F0 range

**Figure 15:**
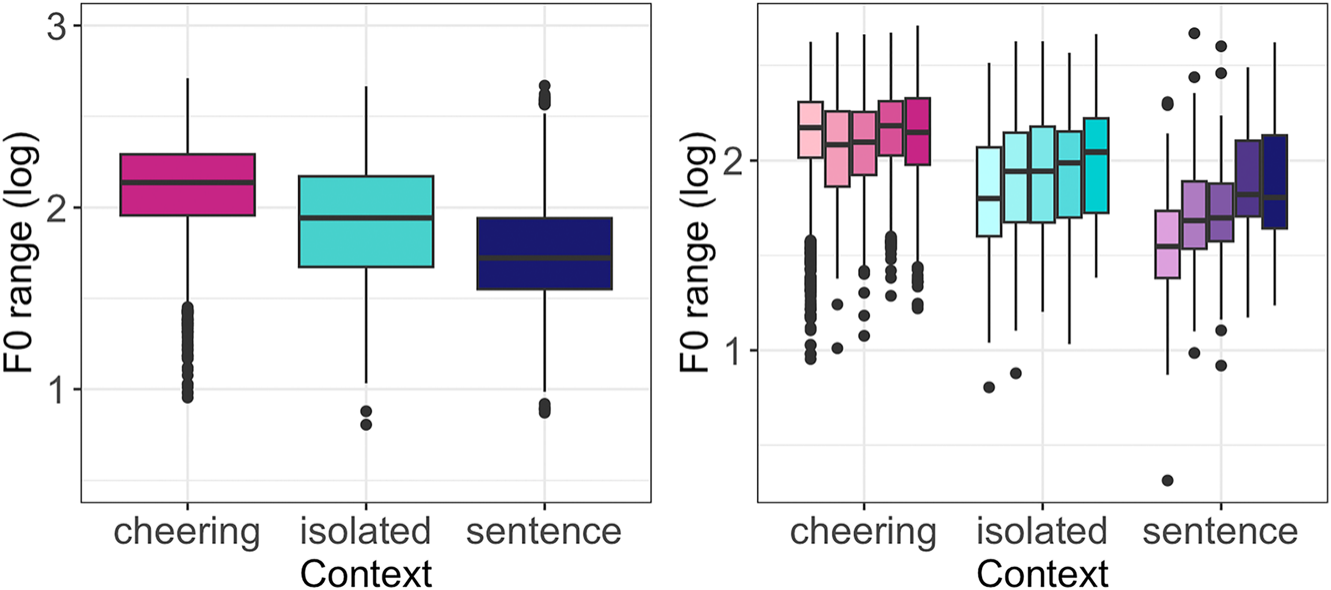
F0 range of words in different contexts (left) split for 1- to 5-syllabic words (right).

Concerning the F0 range, our results reveal again a significant impact of the context type (F(2, 29,6 = 59.35, p < 0.001)). Items produced in cheering showed a significantly larger F0 span in comparison to the items produced in isolation (*b* = 0.22, *t* = 4.44, *p* < 0.001) and sentences (*b* = 0.39 *t* = 9.47, *p* < 0.001), see Figure 15 (left). No significant differences were found between words of different lengths. Figure 15 (right) illustrates the results for different contexts. There was also no signficant difference between male and female speakers.

**Figure 16:**
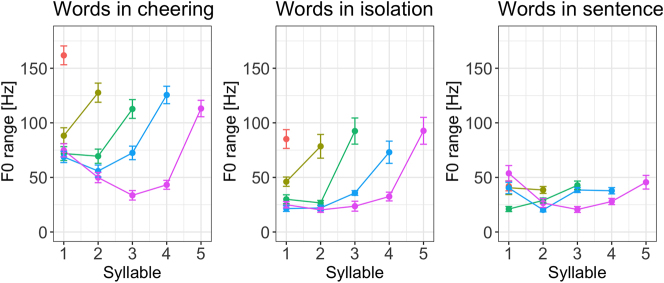
F0 range of syllables for 1–5 syllabic words split for contexts.

If we look at the F0 range of individual syllables across the three contexts it turns out that words produced in cheering had a rather sharp increase from the prefinal to the final syllable in the cheering and isolation context. For words produced in the sentence context a rather mild increase in the F0 range was found mostly in 5-syllabic words, see Figure 16.

**Figure 17:**
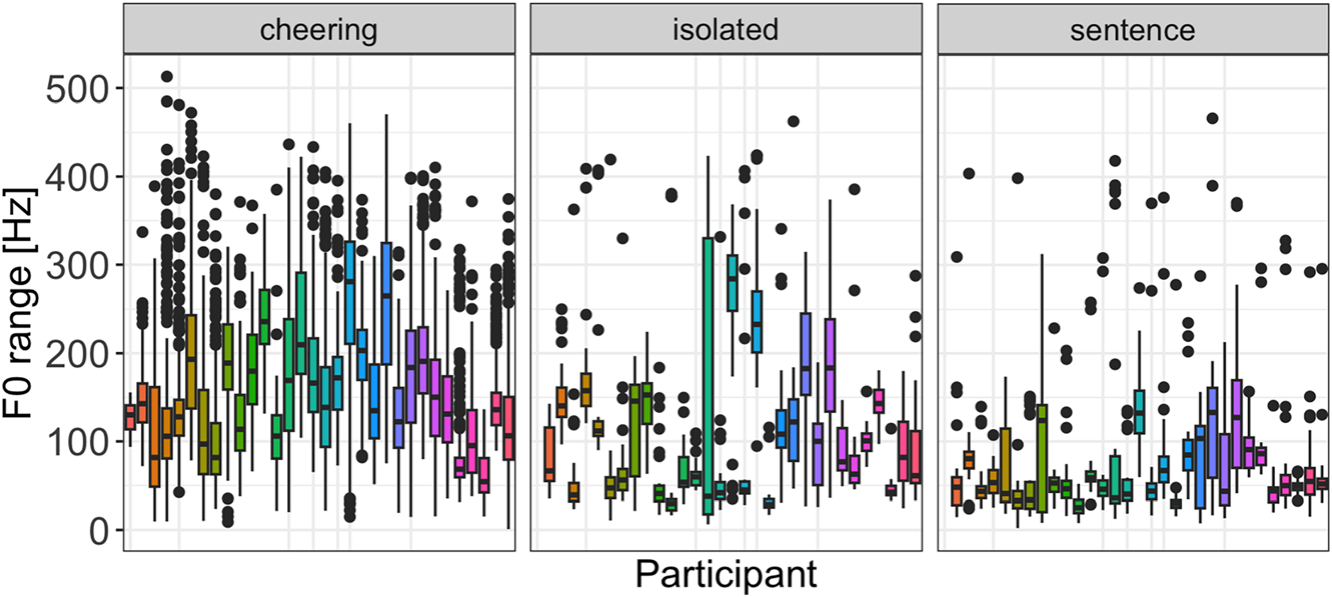
F0 range in different contexts for individual participants.

Finally, it is worth mentioning that the F0 range was subject to a huge inter-speaker variation across all three contexts, whereby it was the cheering context that was characterized by the most extreme variation (Figure 17).

#### 3.6.5 Speech rate

**Figure 18:**
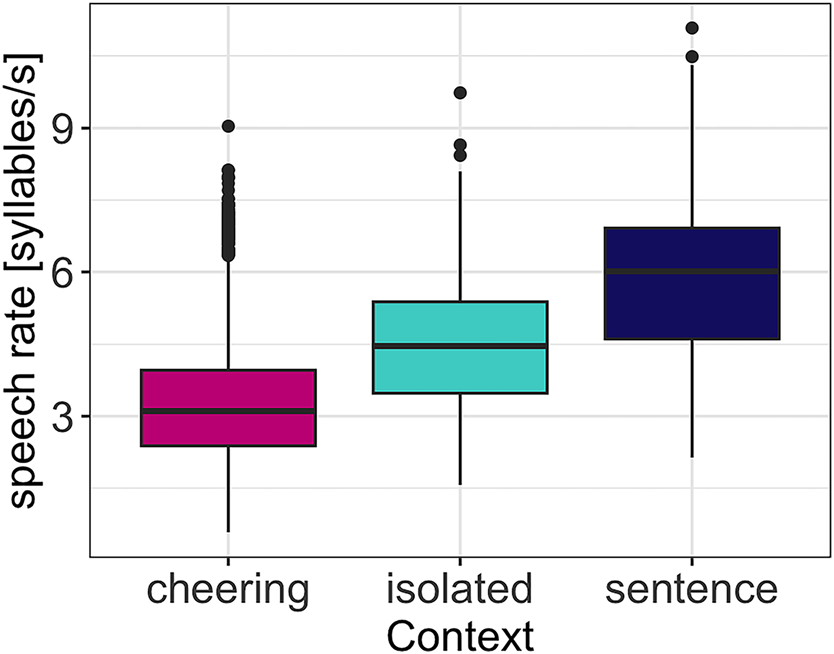
Speech rate in different contexts.

Regarding speech rate, our results reveal that context type (F(2, 29.5) = 133.14, p < 0.001) significantly affected speech rate. Items in the cheering contexts were overall produced with a significantly slower speech rate as compared to the items produced in isolation (*b* = -1.07, *t* = −7.52, *p* < 0.001) and in the sentence context (*b* = −2.40, *t* = −13.99, *p* < 0.001). This is illustrated in Figure 18. Furthermore, longer words were produced with lower speech rate (F(1, 31.4) = 289.71, p < 0.001). Male and female speakers behave differently. Female speakers produced words of the female gender with a higher speech rate (3.63 syllables/s) than words of the male gender (3.50 syllables/sec) whereas this difference was smaller for male speakers (female names: 3.50 syllables/s vs male names 3.43 syllables/sec; interaction: *F*(1, 12,203) = 14.6, p < 0.001). Again, age was not significant. Please note that we did not calculate the speech rate for each word length as was the case for other parameters because we aimed to calculate the speech rate for longer domains (IPU units) that mirror the speech rate more insightfully.

**Figure 19:**
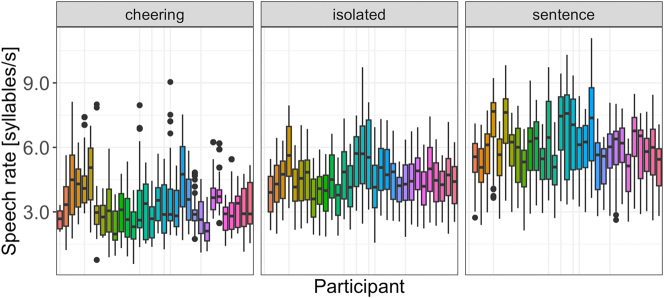
Speech rate in different contexts for individual participants.

As evidenced by the results illustrated in Figure 19 speakers used different speech rates in all three contexts with more extreme values found in the cheering context.

#### 3.6.6 The importance of the acoustic parameters for selecting cheering contours

The random forest analysis compares the relative reliability of different acoustic features in the production of items produced in cheering contexts as opposed to items produced in other contexts, i.e., in the isolation and sentence contexts. In our analysis, the two latter contexts were merged into one “other” context.

**Figure 20:**
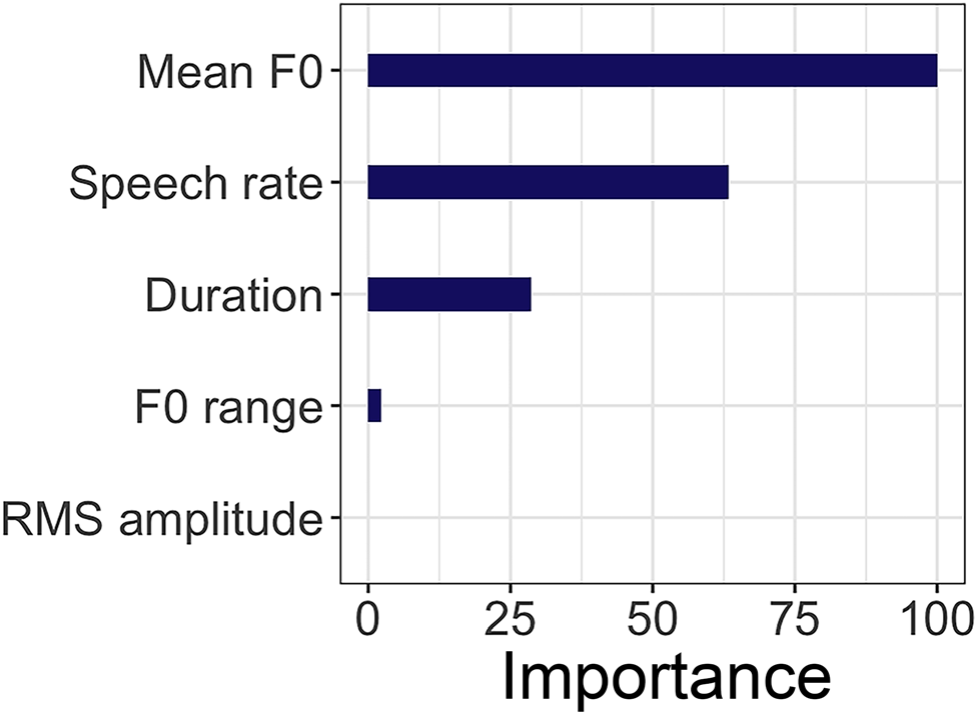
Variable importance of different acoustic features for items in cheering versus other contexts.

The results reveal that F0 is the most important parameter for the selection of cheering contours. It is followed by speech rate and duration. The F0 range seems to have a very small importance in the selection of cheering contours. Finally, the least importance was assigned to the amplitude (at least in our data, but see the discussion in Section 4). Figure 20 illustrates the importance of each acoustic parameter for the selection of cheering items in contrast to items produced in other contexts.

#### 3.6.7 Stress shift

**Figure 21:**
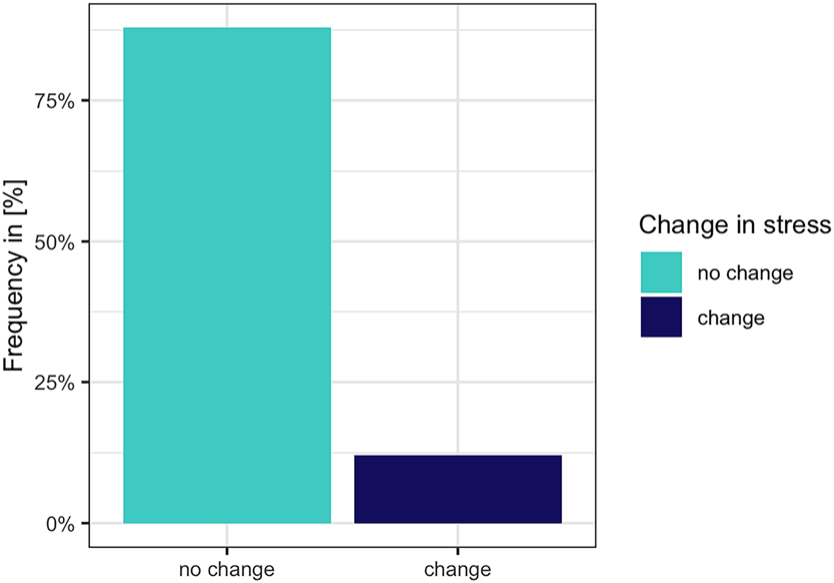
Stress shift in the cheering items.

Before concluding we will also present the results related to stress shift in cheering items. We only annotated three- to five-syllabic words in which the stress could be potentially shifted. If there was a clear difference in the perception of stress deviating from the canonical pattern, i.e. penultimate stress, we marked this as a change. We did not mark the changes if the stress was perceptually evenly spread across the syllables of a word. Thus, only names, in which the stress was clearly heard on the non-penultimate syllable were marked. The analysis was performed by one annotator and randomly checked on a small data subset of another annotator. The results reveal that in only 10.23 % (1,081 items) of the cases, the stress was heard on other syllables than the penultima. In the remaining cases (89.77 %, 10,496 items) the stress did not change. See Figure 21.

Importantly, stress shifts were observed in the productions of only three speakers, with the proportion of affected cases amounting to 42 %, 53.5 %, and 100 %, respectively. As for individual names, the stress shift was relatively evenly distributed, with the following proportions: Johannes (10.6 %), Emanuela (10.1 %), Angelina (9.64 %), Daniela (9.63 %), Bartholomäus (8.34 %), and Alexander (7.3 %).

## 4 Discussion

In this paper, we investigate a use of language rarely discussed before, namely cheering in sport events. We addressed this from a speech act-theoretic perspective, as an act that encourages the athlete or sports team, but also creates a bond between the cheerers. We were, in particular, interested in the prosody of cheering. While this can be investigated in the wild at sport events, we wanted to get detailed information on the prosodic realization of cheering that can only be obtained in the lab. We designed an experiment in which individual participants were asked to cheer an individual runner in a video tape of a long-distance running event, using the name of the runner. The names were varied from one to five syllables.

The outcomes of our investigation elucidate the diverse strategies employed by speakers to fulfil their communicative objectives. Specifically, we discerned four distinct cheering patterns manifested by speakers, each exhibiting unique characteristics:

(i) Separate Production: Participants exhibited a pattern characterized by individually produced names with comparable durations, interspersed with relatively prolonged pauses.
(ii) Syllabic Division: Another observed pattern involved the segmentation of items into syllables, emphasizing a rhythmic articulation.
(iii) Mixed Pattern: A subset of speakers demonstrated a mixed pattern, combining features of both separately produced items and syllabic divisions.
(iv) Singing Pattern: Finally, a distinct cheering pattern emerged, characterized by a melodic or singing quality, incorporating elements of both separately produced items and syllabic divisions.

The second pattern seems to be close to the pattern “emphasis for attention” outlined by Niebuhr (2022: 308). This pattern is based on the reiteration of words, e.g. “go, go” or pitch accents, as. e.g. “eve-ry-sin-gle-day!” and the reiterations are integrated into an overarching prosodic structure which corresponds to our IPU. As noticed by Niebuhr, this class of emphasis-for-attentions is the least investigated in the Kiel Intonation Model (see Niebuhr (2022) for an overview). The fourth pattern could be potentially compared to calling contours in neutral contexts but its F0 is too flat (see Arvaniti et al. 2016 for an overview of calling contours). These identified patterns underscore the variability in cheering strategies employed by speakers during cheering events, highlighting nuanced approaches to achieve communicative efficacy.

Our acoustic analysis revealed that names produced as cheering items are extremely different from those produced in neutral speech mode, either in isolation or embedded in a sentence. Virtually, all parameters we tested significantly differ in dependence on the context.

Results on duration reveal that items produced in the cheering context exhibited the longest duration. This finding contradicts our hypothesis, which predicted that cheering items would be shorter than those produced in other contexts. We expected cheering to be as short as possible to align with the speed of the runners. The results reflected a prolonged duration of the cheering items in line with different types of “roaring” in sports events like soccer; see the live football commentaries analysed by Trouvain (2011). It is noteworthy that these items also demonstrated the highest degree of inter-speaker variability compared with those generated in the isolation and sentence context. Moreover, both prefinal and final syllables exhibited a prolonged duration in polysyllabic words in the cheering and isolation context, but not in the sentence context. We interpret the observed prolongation as a final lengthening, a phenomenon occurring specifically at phrase boundaries in German (Petrone et al. 2017). It was found in the first two contexts but not in the sentence where the target word was followed by another word resulting in the formation of a unified phrase.

We expected a greater intensity in the cheering situation. The rationale for this is that cheerers experience greater arousal in comparison to non-cheerers, and/or iconically express greater support with the exertion of more energy. Our results, i.e., the RMS amplitude confirmed it. Items produced in the cheering context were louder than items produced in the isolated and sentence context. Moreover, the amplitude word profiles differed from each other: the amplitude notably increased towards the end of cheering items but not in the other context. We interpret this finding as due to the well-known non-finality effects where non-final units are produced with increased amplitude (Ladd 1980). Again, the RMS amplitude was characterized by a huge inter-speaker variation. It is possible that variations in the amplitude resulted from the heightened sensitivity of this parameter, necessitating precise control over distance. Unintentional modifications by our speakers could account for discernible differences or their absence, potentially stemming from distance changes to the microphone.

Additionally, despite our efforts to mimic a cheering scenario, similar to cheering at a sports event, the experiment still took place within a controlled laboratory setting. Notably, participants engaged in cheering without the presence of other cheerers, which is unnatural for cheering and might have placed inhibitions on some of them. Furthermore, an increasing intensity is generally expected when the distance from the speaker increases or when someone is far away. This was not the case in our lab when the results were so dependent on the imaginative capabilities of the speakers and their willingness to adapt themselves to a potentially authentic context. In consequence, some speakers did not produce amplitude differences across the contexts. Moreover, as shown in Figure 11 there were quieter realizations of cheering items compared to those produced in other contexts. This was likely due to the fact that cheering items involved greater voice modulation, including variations in intensity, which differed from the relatively stable intensity observed in other contexts. Finally, it should be also stressed that the amplitude measured in [dB] (and not in RMS) showed even a greater variation which affected the results in a way that amplitude differences were only at the level of a statistical tendency (see Żygis et al. 2022). As shown by the random forest analysis the amplitude was the weakest parameter contributing to the selection of cheering items in our data. Regarding amplitude profiles, our results reveal that the amplitude increased in the cheering items towards the final boundaries of words whereas in other contexts it fell or remained stable.

Next, our results point to the fundamental frequency (mean F0 measured in ERB) as being highest in the cheering context as opposed to other contexts. Speakers produced a significantly higher mean F0 when cheering compared to their production of the same words in isolation and frame sentences. An analysis of F0 profiles revealed that in the final part of words, primarily from the penultimate to the ultimate syllable, F0 decreased in the cheering context, in contrast to the isolation context, where it was slightly rising. This difference might be because participants could have produced the latter items as a list (with increasing F0). F0 in the sentences did not show large fluctuation and remained rather stable towards the ends of words.

Our random forest analysis revealed that F0, in our case the higher F0, is the most powerful parameter employed by speakers to produce cheers. This is in line with findings about higher F0 means in the expression of high-arousal emotions, see Bänziger and Scherer (2005). A higher F0 can be seen not only as expressing higher arousal of the speaker but also as the intention to instil higher arousal in the addressee, thereby supporting them in their efforts.

Voße and Wagner (2018) have also found that a higher F0 (median, range, and variation) leads to a higher degree of successful motivation. At the same time, they observed inconsistent results for the variation of intensity, suggesting a more fine-grained analysis of this parameter. Similarly, Niebuhr et al. (2020) reported that a higher F0 level (and range) was used in more charismatic speeches by Steve Jobs as compared to less charismatic speeches by Mark Zuckerberg, but the intensity mean was found to be significantly higher in the investor-oriented speeches held by Steve Jobs; no intensity difference was found in the customer-oriented speeches between the speakers.

We also found differences in speech rate: Names in the cheering context were produced with the slowest speech rate as compared to names produced in other contexts. We did not expect this, as the speakers aimed to motivate the runners to speed up, so one would expect the rate of the motivating speech to be increased as a matter of iconicity. However, greater duration may also convey iconically greater stamina. Closer inspection of the data revealed that speakers very often prolonged the duration of selected vowels and syllables, or they cheered on the runners by syllabifying the names and therefore introducing (very short pauses) between the syllables of a given name (Pattern 2). This is reflected in the duration measurements: The cheering items were significantly longer and showed more variation than those produced in isolation and sentences. This finding leads us to reject our initial hypothesis about the fastest speech rate of cheering items.

It should also be noted that the prosodic features found in the cheering contexts are only seemingly similar to those found in Lombard speech (Anglade and Junqua 1990; Applebaum and Hanson 1990; Applebaum et al. 1996; Hansen 1988; Hanley and Steer 1949; Kobashikawa et al. 2019; Lombard 1911). The increase in fundamental frequency during Lombard speech is a natural and adaptive response to ensure that the speaker’s voice stands out above background noise. By raising the pitch of their voice, speakers can enhance the intelligibility of their speech and make it easier for listeners to understand them in challenging acoustic conditions. The Lombard speech is also characterized by prolonged duration, lower speech rate and increased amplitude, all of which were found for items produced in the cheering context in the present study. Despite these similarities, the considerable variability observed both between and within speakers suggests that these features cannot be attributed to the Lombard effect. The study’s participants were exposed to only two slightly different background noise conditions: one associated with videos of male runners (mean amplitude: 62.81 dB) and another with videos of female runners (mean amplitude 63.17 dB). The long-term average spectra (LTAS) of these two videos are provided in the Appendix (Figures A3 and A4). Notably, the data reveal significant inter- and intra-speaker variability in all parameters, regardless of the video type presented. There was no pattern to be detected as far as the reaction to the two video types is concerned. For instance, regarding the amplitude speakers employed different strategies: higher amplitude when calling male names (female speaker 12: 67.02 dB (male names) versus 66.27 dB (female names); male speaker 26: 70.20 dB (m) versus 69.30 dB (f)), lower amplitude when calling male speakers (female speaker 17: 69.63 dB (m) versus 71.45 dB (f); male speaker 21: 62.34 dB (m) versus 64.73 dB (f)) or almost the same (male speaker 7: 66.77 dB (m) versus 66.20 dB (f), female speaker 10: 68.55 dB (m) versus 68.42 dB (f)). This observation supports the interpretation that the variability in acoustic parameters is more likely due to intentional vocal modulations associated with the act of cheering, rather than a mere reaction to background noise, as seen in the Lombard effect.

It is worth highlighting that we also found differences between male and female participants, with the most striking one being that female speakers produced female names shorter than male ones, whereas no difference was found for male speakers. Additionally, female speakers produced female words with a higher speech rate than words of male gender.

Finally, our analysis of stress distribution revealed that speakers relatively rarely changed their lexical stress (approximately 10 %). In the remaining cases, they either kept the stress on the penultimate syllable or the stress was evenly distributed over the syllables of a given item.

## 5 Conclusions

We have presented a first study of the prosodic realization of a speech act that belongs to the class of cheering, namely the encouragement of athletes that compete in sports events, more specifically, cheerings that use the name of the addressee. The study investigated cheering understood as *inciting support* (“Anfeuern”) during an ongoing event, rather than rejoicing after an achievement. This type of cheering is characterized by higher F0 and wider F0 range, increased loudness, longer durations, and slower speech rate, which distinguish it clearly from other types of cheering such as celebratory or triumphant cheering. The cheering items in our study were also longer, louder, and produced with a higher F0, larger F0 span and lower speech rate in comparison to the same items produced in isolation and embedded in sentences. The word profiles especially concerning the increased duration of items and higher F0 range were more similar in the cheering context and isolation as opposed to the items embedded in sentences. We assume that this similarity follows from the fact that cheering items are isolated items, often creating a prosodic phrase on their own and having a different pragmatic function.

Our study of cheering contours reveals that speakers follow different strategies to meet their communicative goals, i.e., to motivate sports competitors (in our case, long-distance runners). We identified four different cheering patterns executed by speakers: (i) separately produced items of similar duration with relatively longer pauses, (ii) division of items into syllables, (iii) mixed pattern of (i) and (ii), and finally (iv) singing pattern, again mixed with (i) and (ii).

All speakers repeated their cheers by uttering the name several times. The motivation for this is evidently to express continued support for an ongoing event. Also, repetition may convey greater stamina to the addressee. In our study, the repetitions were nearly always rhythmically spaced out in equal intervals. In our view, this may support the rhythmical movements of the athlete, by aligning the speech signal to these movements. It also might convey stamina and the determination of not giving up in an iconic way.

## Figures and Tables

**Figure 1A:**
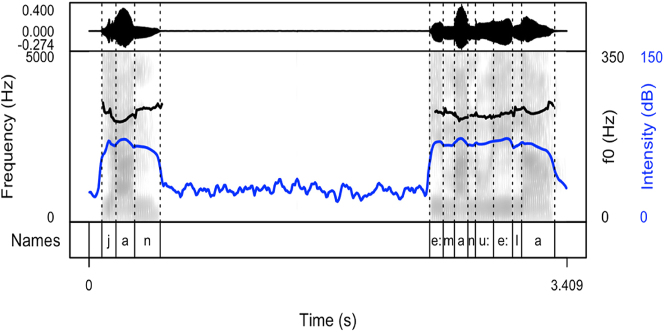
Spectrogram and oscillogram of the name “Jan” produced in an isolated context.

**Figure 2A:**
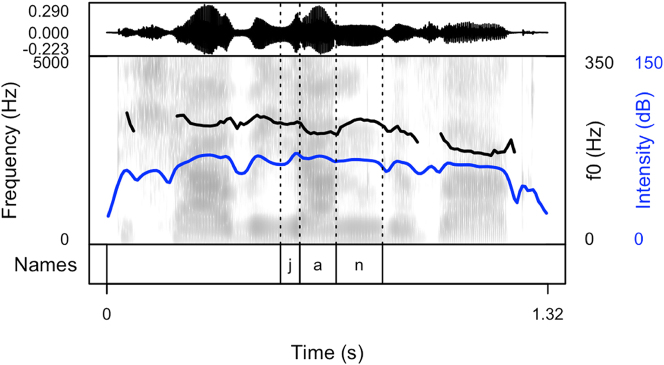
Spectrogram and oscillogram of the name “Jan” produced in a sentence context.

**Figure 3A:**
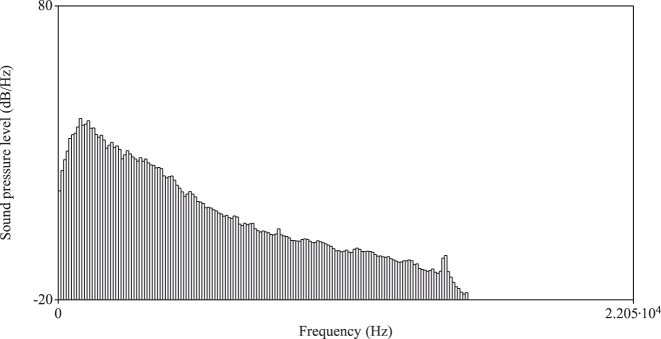
LTAS of the background noise in a video labeled with female names.

**Figure 4A:**
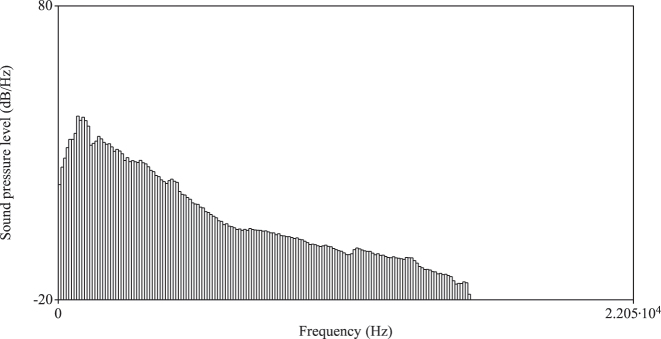
LTAS of the background noise in a video labeled with male names. Long-Term Average Spectrum (LTAS) of a video featuring male-named participants. The spectrum illustrates the average distribution of acoustic energy across frequencies throughout the recording. Elevated energy in the lower frequency bands reflects the predominance of background noise such as ambient crowd sounds and environmental hum.

## Appendix

See Figures 1A–4A.
